# Dual role of intratumoral microbiota: From colorectal cancer progression to therapeutic opportunities

**DOI:** 10.1515/jtim-2026-0042

**Published:** 2026-06-13

**Authors:** Anqi Lin, Xiuhui Fang, Lingxuan Zhu, Weiming Mou, Peng Luo

**Affiliations:** Department of Oncology, Zhujiang Hospital, School of Stomatology, Southern Medical University; Donghai County People's Hospital (Affiliated Kangda College of Nanjing Medical University), Lianyungang, Jiangsu Province, China; Department of Urology, Shanghai General Hospital, Shanghai Jiao Tong University School of Medicine, Shanghai, China

## Introduction

Colorectal cancer (CRC) ranks as the third most commonly diagnosed malignancy and the second leading cause of cancer-related mortality worldwide. The microbiota plays a pivotal role in the initiation and progression of CRC. However, conventional studies have primarily examined correlations between the overall gut microbiota and host cancer, thereby limiting insights into the complex interactions between microbes and the tumor microenvironment (TME). With advances in next-generation sequencing technologies and high-resolution imaging, researchers have identified distinct populations of intratumoral microbes within the TME, thereby ushering in a new era that transitions the focus from gut microbiota to the exploration of intratumoral microbial ecosystems.^[1,2]^ This perspective will systematically discuss the compositional characteristics, underlying mechanisms, and potential translational significance of intratumoral microbiota in CRC.

## Compositional characteristics and spatial heterogeneity of intratumoral microbiota

The intratumoral microbial communities in CRC demonstrate unique compositional characteristics and marked spatial heterogeneity. The microbial composition of tumor tissue differs substantially from that of adjacent normal tissues. For instance, in one foundational study, the intratumoral microbiota was reported to be predominantly composed of *Firmicutes* (63.46%) and *Bacteroidetes* (12.77%).^[1]^ By contrast, the microbiota of adjacent normal tissues is dominated by *Pseudomonas* and *Escherichia-Shigella*, which represent the predominant taxa.^[1]^ Moreover, the presence of intratumoral microbiota is closely associated with pathological stage, with detection rates of *F. nucleatum* in adenoma, stage 0, stage I/II, and stage III/IV being 5.9%, 26.1%, 35.1%, and 81.8%, respectively.^[1]^ Further investigations have revealed that intratumoral microbiota exhibit heterogeneous spatial distribution patterns. Bacterial communities preferentially colonize tumor microniches characterized by lower vascularization and prominent immunosuppression, and are closely associated with malignant cells exhibiting lower Ki-67 expression levels. ^[3]^ The fundamental biological significance of this spatial heterogeneity lies in its role as a critical driver of TME heterogeneity. Intratumoral microbiota can alter the biological properties of distinct cellular compartments, modulate immune cell and epithelial cell functions at the single-cell level, influence antitumor immunity and cancer epithelial cell migration, and consequently drive the evolution of tumor heterogeneity in patients.

## The role of intratumoral microbiota in CRC development

**Figure 1:**
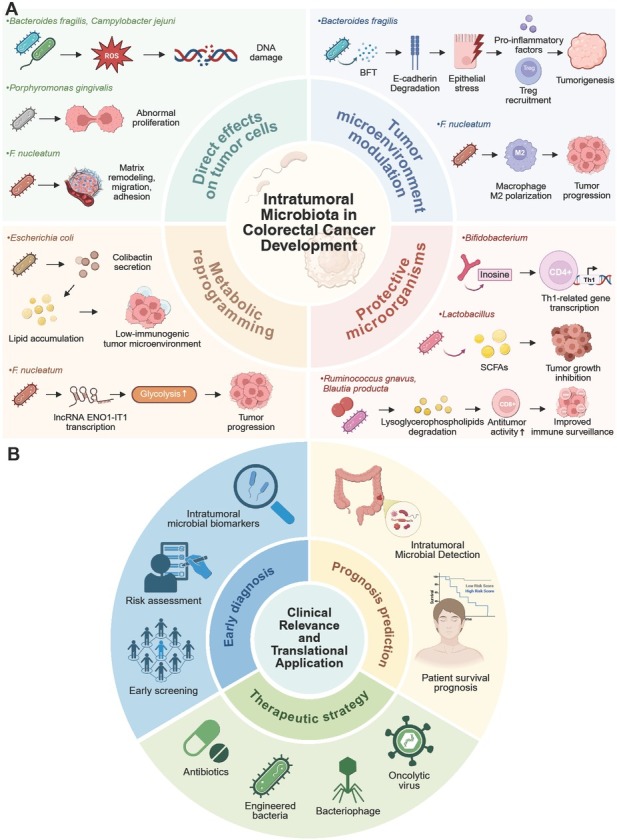
Intratumoral microbiota in colorectal cancer—their roles in tumor development, clinical relevance, and translational applications. (A) Intratumoral microbiota play a dual role in colorectal cancer development. Oncogenic microorganisms, such as *Bacteroides fragilis*, *Escherichia coli*, and *Fusobacterium nucleatum*, can promote the initiation and progression of colorectal cancer by directly interacting with tumor cells, modulating immune responses, and inducing metabolic reprogramming. In addition, certain intratumoral microorganisms, such as *Bifidobacterium*, *Lactobacillus*, *Ruminococcus*, and *Faecalibacterium prausnitzii*, exert tumor-suppressive effects by inhibiting tumor cell proliferation or modulating immune cell functions. (B) Intratumoral microbiota hold significant potential for early diagnosis, prognostic prediction, and therapeutic intervention in colorectal cancer. They may serve as promising biomarkers for risk assessment and early screening. Moreover, alterations in microbial abundance and composition are closely associated with patient survival, thereby underscoring their prognostic significance. Furthermore, microbiota-based intervention strategies, including antibiotics, engineered bacteria, bacteriophages, and oncolytic viruses, provide novel avenues for precision medicine and personalized interventions in colorectal cancer. Created in BioRender. ROS: reactive oxygen species; BFT: *Bacteroides fragilis* toxin; Treg: regulatory T cell; SCFAs: short-chain fatty acids.

Intratumoral microbiota play a dual regulatory role in the development of CRC. On the one hand, they may promote tumor initiation and progression. On the other hand, certain intratumoral microbes exert protective effects (Figure 1A).

### Direct effects on tumor cells

Intratumoral microbiota contribute to CRC progression by inducing genomic instability in tumor cells and activating multiple oncogenic signaling pathways. *Bacteroides fragilis* and *Campylobacter jejuni* secrete toxins and other metabolites that induce excessive reactive oxygen species production, thereby causing DNA damage.^[4]^ Intracellularly colonized *Porphyromonas gingivalis* can activate the mitogen-activated protein kinase (MAPK)/extracellular regulated protein kinases (ERK) pathway through its potent virulence factor gingipain, thereby promoting CRC cell proliferation.^[5]^ Pathway-level differential expression analyses indicate that the presence of intratumoral *F. nucleatum* leads to upregulation of growth factor signaling pathways, such as epidermal growth factor receptor (EGFR) and platelet-derived growth factor (PDGF), as well as the epithelial-mesenchymal transition (EMT) and nuclear factor kappa B (NF-κB) signaling pathways, which are associated with cancer cell migration; however, the specific molecular mechanisms remain to be further elucidated.^[3]^

### Regulation of the TME

Intratumoral microbiota have the capacity to regulate cytokine secretion and immune cell functions within the TME, thereby facilitating tumor initiation and progression. The *Bacteroides fragilis* toxin, secreted by *Bacteroides fragilis*, promotes E-cadherin degradation, triggers epithelial cell stress responses, and subsequently induces the excessive release of pro-inflammatory mediators, further enhancing regulatory T-cell recruitment, ultimately contributing to tumorigenesis. *F. nucleatum* promotes tumor progression by inducing macrophage polarization toward the M2 phenotype *via* a TLR4-dependent mechanism, thereby establishing an immunosuppressive TME.^[1]^

### Metabolic reprogramming

Intratumoral microbiota can induce tumor metabolic reprogramming through the reshaping of metabolic pathways, thereby sustaining and promoting tumor progression. Colibactin produced by *Escherichia coli* induces lipid metabolic dysregulation, leading to the formation of a microenvironment enriched in phosphatidylcholine but characterized by low immunogenicity. *F. nucleatum* activates the transcription of lncRNA ENO1-IT1, which regulates histone modifications and thereby enhances cancer cell glycolysis and promotes CRC progression.^[5]^

### Protective microorganisms

Beyond their pro-tumorigenic effects, certain intratumoral microbes exhibit protective or tumor-suppressive properties, which can be mediated through either their direct presence and activity within tumor tissues or through systemic modulation of immune responses in the gut. Tissue-resident *Ruminococcus gnavus* and *Blautia producta* provide early local anticancer protection by degrading lysophosphatidylcholine, thereby relieving its inhibitory effects on CD8^+^ T cell activity.^[5]^ Furthermore, inosine produced by *Bifidobacterium* induces transcriptional differentiation of Th1 cells in the gut-associated lymphoid tissue mucosa and enhances antitumor immunity *via* A2AR signaling pathways.^[6]^ *Lactobacillus* inhibits tumor growth by increasing short-chain fatty acid production, thereby altering the intestinal metabolic profile and microbial composition.^[4]^

## Clinical relevance and translational applications of intratumoral microbiota in CRC

Intratumoral microbiota in CRC exhibits significant translational potential in early diagnosis, prognostic prediction, and the development of personalized therapeutic strategies, thereby offering both a theoretical foundation and practical opportunities to advance precision oncology (Figure 1B).

### Early diagnosis

The tumor-specific enrichment of certain microbial taxa confers upon the intratumoral microbiota the potential to serve as novel biomarkers for early CRC detection. The enrichment of *Escherichia coli* in patients with familial adenomatous polyposis- a hereditary condition that predisposes to CRC- suggests its potential role in early carcinogenesis, possibly through genotoxin production, underscoring its utility as a microbial biomarker for CRC risk assessment.^[7]^ Studies have revealed substantial differences in the plasma microbiota composition between CRC patients and healthy individuals, with 28 taxa—including *Ruminococcus torques* and *Prevotella intermedia*—identified as potential noninvasive biomarkers for the early screening of CRC.^[4]^

### Predicting prognosis

A substantial body of evidence suggests that variations in the abundance and composition of intratumoral microbiota are strongly associated with patient prognosis. Compared with patients who are negative for *F. nucleatum*, those exhibiting either low or high levels of this bacterium show a significantly elevated risk of mortality.^[7]^ Furthermore, compared with the *Firmicutes*/*Bacteroidetes* subtype, stage II/ III patients classified under the *Fusobacterium*/oral pathogens subtype or the *Escherichia*/*Pseudescherichia*/*Shigella* subtype exhibit significantly shorter overall survival.^[8]^ Although multiple studies have reported associations between intratumoral microbial abundance and patient survival outcomes, these findings require further validation in multivariable models that incorporate clinical confounding factors, such as tumor stage, treatment history, and molecular subtypes. Future studies should conduct prospective, standardized, multicenter cohort investigations to systematically assess the independent prognostic value of microbial signatures, thereby enhancing their clinical interpretability and translational potential.

### Therapeutic strategies based on intratumoral microbiota

Interventions tailored to patients’ microbial profiles hold considerable promise for advancing multimodal combinatorial cancer treatment strategies.^[7,9]^ Currently, therapeutic modalities that have advanced to clinical validation stages primarily include antibiotics and oncolytic viruses. Antibiotics can eliminate pro-tumorigenic or drug resistance-promoting microbial populations, thereby reshaping the intratumoral microenvironment; oncolytic viruses exert dual functions of lysing tumor cells and enhancing host antitumor immunity.^[1]^ Meanwhile, researchers are actively exploring emerging novel therapeutic strategies. Engineered bacteria can serve as therapeutic platforms for delivering anticancer drugs and activating antitumor immune responses; bacteriophages can not only specifically target drug-resistant or oncogenic strains but also achieve immune cell recruitment through engineered modifications.^[1]^ For instance, recent studies have demonstrated that engineered *Escherichia coli* can selectively colonize tumor tissues and locally deliver therapeutic molecules, thereby enhancing tumor-killing efficacy. Furthermore, research has reported that nanoparticle-mediated delivery of melittin can effectively eliminate the key oncogenic bacterium *F. nucleatum* within tumors, reshape the TME, and significantly enhance anticancer therapeutic efficacy.^[10]^ These findings further underscore the promising potential of precision microbiota modulation as a next-generation therapeutic strategy.

## Challenges

Research on intratumoral microbiota in CRC still faces numerous challenges. First, the inherently low biomass of intratumoral microbiota and their high susceptibility to contamination complicate microbial isolation, purification, and data interpretation, thereby constraining the reliability and reproducibility of research.^[5]^ Second, current detection technologies remain constrained by limited sensitivity and spatial resolution, which impede the in-depth elucidation of dynamic interactions between heterogeneous intratumoral microbiota and the TME.^[3]^ Moreover, systematic investigations into host-microbiota interaction mechanisms and their causal relationships remain limited.^[1]^ Finally, microbiota-based therapeutic interventions continue to require rigorous evaluation and comprehensive optimization to ensure both safety and efficacy.^[7]^ Notably, the absence of standardized protocols for sample collection, processing, sequencing, and bioinformatics analysis in current studies hinders cross-validation and consistency verification of research findings; future investigations should prioritize establishing unified methodological frameworks and conducting large-scale multicenter prospective cohort studies to overcome this limitation.

## Future perspectives

The central task of future research on intratumoral microbiota in CRC is the coordinated advancement of multimodal technology integration, the comprehensive elucidation of causal mechanisms, and the effective translation into clinical practice. First, the optimization of sample collection and detection methods, combined with the integration of multi-omics and artificial intelligence technologies, is essential for constructing a high-resolution microbiota-host interaction network. Secondly, existing research on the mechanisms of host-microbiota interactions and their causal relationships remains insufficient. Future efforts should focus on developing *in vivo* and *in vitro* visualization platforms, functional validation models, and dynamic tracking technologies to elucidate the causal mechanisms by which key microorganisms influence tumorigenesis and progression. Furthermore, microbiota-based therapeutic intervention strategies require rigorous evaluation and comprehensive optimization with regard to safety and efficacy. Of particular interest, recent studies have revealed potential tumor-promoting interactions between the nervous system and microbiota, including modulation of the TME through regulation of local immune responses or metabolic pathways, although direct evidence in CRC remains lacking. Future research should further explore the potential mechanisms of neuro-microbiota interaction networks in CRC progression and immune regulation, while examining the possible cross-recognition relationships between intratumoral microbial antigens and CRC neoantigens, thereby providing critical insights into novel mechanisms by which microbiota participate in tumorigenesis and development.
